# Genome-Wide Analysis of the Cytochrome P450 Gene Family Involved in Salt Tolerance in *Gossypium hirsutum*

**DOI:** 10.3389/fpls.2021.685054

**Published:** 2021-12-02

**Authors:** Kangtai Sun, Hui Fang, Yu Chen, Zhimin Zhuang, Qi Chen, Tingyu Shan, Muhammad Kashif Riaz Khan, Jun Zhang, Baohua Wang

**Affiliations:** ^1^School of Life Sciences, Nantong University, Nantong, China; ^2^Key Laboratory of Cotton Breeding and Cultivation in Huang-Huai-Hai Plain, Ministry of Agriculture and Rural Affairs, Cotton Research Center of Shandong Academy of Agricultural Sciences, Jinan, China; ^3^Plant Breeding and Genetics Division, Nuclear Institute for Agriculture and Biology, Faisalabad, Pakistan

**Keywords:** P450, gene family, differentially expressed genes, salt tolerance, cotton

## Abstract

Plant cytochrome P450 (P450) participates in a wide range of biosynthetic reactions and targets a variety of biological molecules. These reactions lead to various fatty acid conjugates, plant hormones, secondary metabolites, lignin, and various defensive compounds. In our previous research, transcriptome analysis was performed on the salt-tolerant upland cotton “Tongyan No. 1.” Many differentially expressed genes (DEGs) belong to the P450 family, and their domains occur widely in plants. In this current research, P450 genes were identified in *Gossypium hirsutum* with the aid of bioinformatics methods for investigating phylogenetic relations, gene structure, *cis-*elements, chromosomal localization, and collinearity within a genome. qRT-PCR was conducted to analyze P450 gene expression patterns under salt stress. The molecular weights of the 156 P450 genes were in the range of 5,949.6–245,576.3 Da, and the length of the encoded amino acids for all the identified P450 genes ranged from 51 to 2,144. P450 proteins are divided into four different subfamilies based on phylogenetic relationship, gene structure, and chromosomal localization of gene replication. The length of P450 genes in upland cotton differs greatly, ranging from 1,500 to 13,000 bp. The number of exons in the P450 family genes ranged from 1 to 9, while the number of introns ranged from 0 to 8, and there were similar trends within clusters. A total of 31 *cis-*acting elements were identified by analyzing 1,500 bp promoter sequences. Differences were found in *cis-*acting elements among genes. The consistency between qRT-PCR and previous transcriptome analysis of salt tolerance DEGs indicated that they were likely to be involved in the salt tolerance of cotton seedlings. Our results provide valuable information on the evolutionary relationships of genes and functional characteristics of the gene family, which is beneficial for further study of the cotton P450 gene family.

## Introduction

With the continuous expansion of activities related to human survival and the rapid development of industrial activities, earth’s environments are continuously deteriorating, which has led to great challenges in the development of agriculture. Land salinization is one of the important problems facing agricultural production. Salt stress reduces water uptake by the root system and leads to internal dehydration, and salt accumulation affects metabolic processes, especially in photosynthetic cells. Damage to plants can occur when transport tissues carry too much salt (Munns, 2020). It was reported that after cotton was exposed to salt stress, superoxide dismutase (SOD), glutathione reductase (GR), and peroxidase (POD) activity increased with the increase of salt concentration; meanwhile, net photosynthetic rate and stomatal conductance decreased with salt stress, and leaf chlorophyll content decreased significantly (Meloni et al., 2003).

Plant cytochrome P450 (P450) participates in a wide range of biosynthetic reactions and targets a variety of biological molecules. These reactions lead to various fatty acid conjugates, plant hormones, secondary metabolites, lignin, and various defensive compounds (Schuler and Werck-Reichhart, 2003; Tang et al., 2016), e.g., CYP706B1 and CYP82D113 are involved in the biosynthesis of gossypol in plants (Ma et al., 2016; Teh et al., 2017). CYP71AV1 is involved in the biosynthesis of artemisinin. Plant genome annotation shows that P450 genes account for up to 1% of plant genes. The number and diversity of P450 genes are partly responsible for the presence of multiple biologically active compounds (Mizutani and Sato, 2011).

P450 is the largest enzyme family that supports plant metabolism to date and is representative of metabolic structure and evolution. It is a very useful tool for discovering the basic functions of plant metabolism and guiding gene function research (Du et al., 2016; Ghosh, 2017; Li and Wei, 2020). With the development of biotechnology in recent years, a series of factors related to adversity have been identified (Gong et al., 2020; Wang et al., 2020), and the P450 family is widely studied.

In the P450 family, some genes are copied in large numbers (Nelson and Werck-Reichhart, 2011), e.g., genes involved in the phenylalanine pathway. Genes in the P450 family have been verified to be involved in the biosynthesis of jasmonic acid (JA) in plants such as soybeans and wheat, with locations in stems and/or roots (Yan et al., 2016). Studies have also shown that P45094B3 exerts a negative feedback control effect on JA-Ile (jasmonic acid and isoleucine) levels (Koo et al., 2011), and it plays a key role in the weakening of the jasmonate response. Biosynthetic P450 is used in the synthesis of lignin intermediates, sterols, terpenes, flavonoids, isoflavonoids, furanocoumarins and various other secondary plant products, in which it plays a vital role (Schuler and Werck-Reichhart, 2003; Carelli et al., 2011; Nelson, 2011). Some subfamily members are better retained. P450 genes also participate in the primary metabolism of plants involved in the biosynthesis of essential sterols and steroid hormones (Mills et al., 2018). They may even perform chemical defense against specific pathogens in specific species (Schuler, 2010).

Cotton contains gossypol and many terpenoids (Qian and Wang, 1984; Tian et al., 2016). Many terpenoids are used as signal molecules, participating in the interaction between organisms or as defensive compounds that protect plants and resist pathogen infection and animal ingestion (Pompon et al., 1996; Wang et al., 2004; Sunilkumar et al., 2006). Semiterpenoids have great complexity and diversity. They are closely related to the diverse enzyme genes, including P450, associated with secondary metabolic pathways (Sunilkumar et al., 2006). Studies have shown that RNA interference is used to inhibit the expression of CYP82D109 in upland cotton. The accumulation of related compounds can be detected in transgenic plants (Zhao et al., 2018). When virus-induced gene silencing (VIGS) inhibits the expression of CYP82D113 in cotton plants, the contents of gossypol and hemigossypolone decrease significantly (Ma et al., 2016). To date, the analysis of the gossypol biosynthetic pathway has not been completed. The functions of the four enzymes, including sesquiterpene synthase CDNC, short-chain alcohol dehydrogenase DH1 and two cytochrome P450 monooxygenases, have been identified (Bathe and Tissier, 2019). In biosynthesis, the hydrocarbon backbone produced by terpene synthase is usually oxidized by cytochrome P450 oxidase. In cotton, gossypol mainly accumulates in the epidermis of roots and aboveground tissues (leaves, stems, flowers, bolls, and seeds) (Guo et al., 2009). The discovery of key genes in the P450 family is helpful for research on stress tolerance in various species.

In our previous research, transcriptome analysis was performed on the salt-tolerant upland cotton line “Tongyan No. 1.” A batch of differentially expressed genes (DEGs) were obtained, some of which belong to the same P450 family. P450 belongs to a multigene family. To fully understand the gene structure and protein function of the P450 family in the upland cotton genome, bioinformatics methods were used to analyze the number of P450 genes in upland cotton and its gene structure, chromosome location, phylogeny, and expression of salt tolerance. The results of this research will help reveal the salt stress response mechanism in cotton.

## Materials and Methods

### Cotton Material

A salt-tolerant upland cotton strain, “Tongyan No. 1,” was used for the following process. Plump and similarly sized seeds were selected and sterilized after dipping in dilute hydrochloric acid, and then they were planted in flowerpots filled with a mixture of nutrient soil and vermiculite at a ratio of 3:1 and cultivated in a greenhouse at a temperature of 23°C with a light:dark ratio of 16:8 h. When the second euphyll was fully expanded, the cotton plants were divided into the control and experimental groups. The control group was watered with 250 ml water solution, while the experimental group was watered with 250 mmol NaCl of the same volume every afternoon (Shaheen et al., 2012). Leaves were collected from the two cotton groups after 2 days in the treatments and stored in an ultralow temperature refrigerator at −80°C for RNA extraction and transcriptome sequencing.

### Identification of P450 Family Members in Upland Cotton

The genome data of upland cotton were obtained from the Cotton Research Institute of Nanjing Agricultural University^1^. The Hidden Markov Model (HMM) database (protein families database of alignments) and HMM. The Pfam database^2^ was used to download the seed file (PF00067) of the P450 gene. HMMER 3.0 software and the local BLASTP program were applied to compare and search for sequences containing the P450 protein domain. After the screening, the protein sequences were uploaded to the online software Pfam, the smart database (Simple Modular Architecture Research Tool, SMART^3^) and the Conserved Domain Database (Conserved domain database, CDD^4^), and sequence alignment and analysis were carried out to remove unannotated genes and redundant sequences. Afterward, the gene IDs containing the P450 domain and its corresponding sequence were obtained. ExPASy Proteomics Server software^5^ was used to analyze the amino acid sequence of all members of the cotton P450 gene family and calculate the amino acid length and isoelectric point.

### Construction of a Phylogenetic Tree of Upland Cotton P450 Family Proteins and Analysis of Conserved Protein Motifs

The MEGA program (MEGA7) was used to perform multiple sequence alignments of the obtained genes (Kumar et al., 2016). Based on the alignment results, an evolutionary tree was constructed with the adjacency joining algorithm. A bootstrap test (replications) with 1,000 iterations was performed. The obtained evolutionary tree was further modified using the Evolview^6^ website. Meme software^7^ was applied to carry out motif analysis based on the protein sequences of P450 family genes. The shortest motif length was six base pairs, and the greatest motif length was 50 base pairs.

### Gene Structure and *Cis-*Element Analysis of P450 Family Genes

The location information of all the P450 family genes, including the exons, introns, and UTR location information on the chromosome, were available in the reference genome of *Gossypium hirsutum* L. acc. TM-1. dataset. A gene structure diagram (Hu et al., 2015) was drawn using the online website GSDS^8^. To explore the regulation of gene expression, 1.5 kb sequences upstream of ATG in all the P450 family genes were extracted to analyze the *cis-*elements of the genes. Plant CARE (*Cis-*Acting Regulatory Element^9^) was used to further analyze the *cis-*elements, and the *cis-*element information obtained was mapped through the GSDS online website.

### Chromosomal Localization of Genes

MapChart software was applied to analyze and draw the chromosome location map, and AI (Adobe Illustrator CS6) software was used to visualize the chromosome position map.

### Collinearity Analysis Within the Genome

All the upland cotton protein sequences were BLAST compared by MCSCANX software, and collinear genes were found throughout the genome. The collinearity of genes in the gene family was discussed, and the gene family circle was drawn by MCSCANX software with special annotation.

### Gene Expression Pattern Analysis of the P450 Gene Family Combined With the Salt Tolerance Transcriptome

P450 family genes were confirmed to be associated with salt tolerance in a previous study on the salt-tolerant transcriptome of the upland cotton ‘‘Tongyan No. 1.’’ The expression heatmap of all the P450 family genes in upland cotton was drawn with the heatmap website^10^, with units of FPKM (fragments per kilobase of exon model per million mapped fragments, that is, the fragments mapped per million transcribed per thousand base length).

### Quantitative Analysis of Candidate Gene Expression

Total RNA was extracted from cotton leaves in various stages after salt treatment of “Tongyan No. 1” using Aidlab’s EASYspin polysaccharide polyphenol plant total RNA extraction kit. Primer5 software was used to design primers for qRT-PCR experiment (Table 1). The M5 Super plus kit purchased from Beijing Polymer Company was used to synthesize first-strand cDNA. Quantitative real-time polymerase chain reaction (qRT-PCR) was carried out in triplicate for each sample using a 2*M5 HIPer Real-time PCR Supermix (SYBRGreen) kit with an ABI 7500 real-time PCR system. The upland cotton *GhUBQ7* (*DQ116441*) gene was used as a control for normalization between samples. Relative transcript levels were calculated using the comparative threshold cycle method (Livak and Schmittgen, 2001).

**TABLE 1:** Primers of qRT-PCR for candidate genes.

Gene ID	Forward primer	Reverse primer
Gh_D04G1016	TGTCGTTCAAGCCAGAGA	GGTGAAGCAATGAGCCTAA
Gh_D05G0425	GTTCAGCAACGACATCCT	ACCTCCTATCTCCACTTCTT
Gh_D12G0534	CTACACTTATCTCCTCGTCTT	CTTCATTCGTCACTTCATCC
Gh_A09G0463	AAGTCCAAGTGCTACAATCA	GCTCCTGCCAAGTTAGTAA
Gh_D04G1716	GTGACTTAGCAACCAACATT	CATCAAGCCAACCGAGAA
Gh_A10G2124	GCCACATCATACCTTCACT	ACCGACTCACTTCTTCATC

## Results

### Identification of Cotton P450 Gene Family

Using domain information (PF00064) obtained from domain prediction to identify members of the whole cotton genome, more than 300 genes were obtained. After comparison of the CDD, SMART, and other databases, 156 pairs of genes were identified as belonging to the P450 gene family. Then, the physicochemical properties of the amino acid sequences of cotton P450 gene family members were analyzed (Supplementary Table 1). The results showed that the molecular weight of the 156 P450 genes was in the range of 5,949.6–245,576.3 Da. The length of the encoded amino acids for all the identified P450 genes ranged from 51 to 2,144, and the theoretical isoelectric point size of the coding protein ranged from 4.15 to 10.97, with an average of 10.23, which is strongly alkaline.

### Phylogenetic Analysis and Conservative Motif Analysis of the P450 Gene Family

**FIGURE 1:**
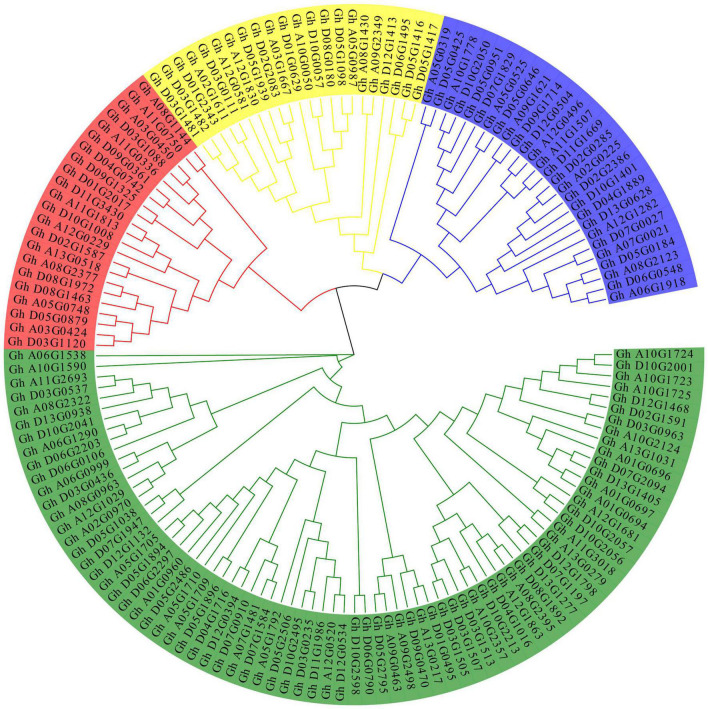
Phylogenetic tree of the P450 family protein sequences in upland cotton.

**FIGURE 2:**
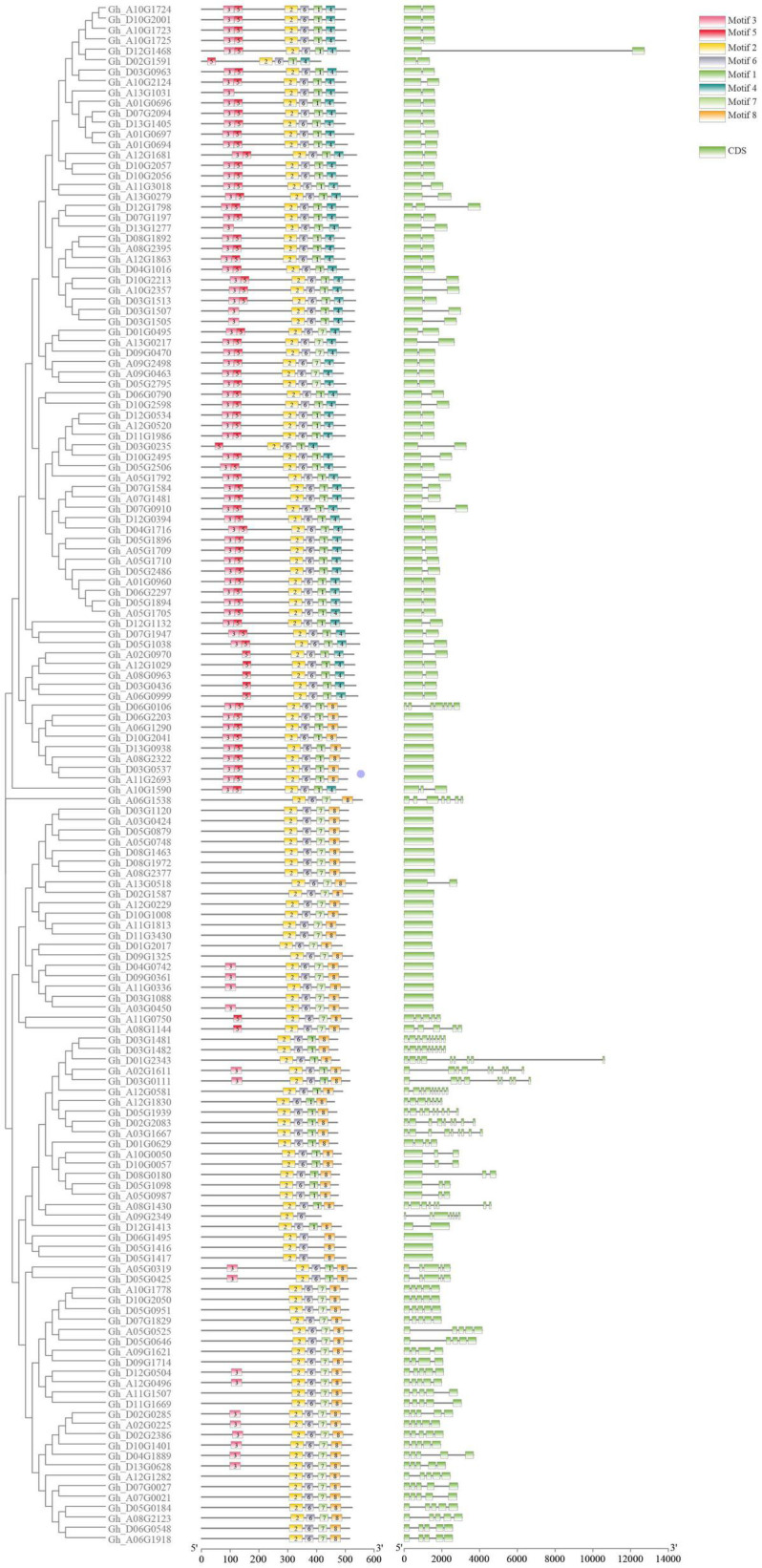
Gene structure diagram of the P450 family in upland cotton. The three columns in the diagram are phylogenetic tree, motif, and exon in order from left to right.

To analyze the evolutionary relationship between the various members of the P450 family, ClustalW in MEGA7 software was used to carry out alignment for the 156 obtained protein sequences, and then the sequences with genetic distances that were too long were removed. A rootless phylogeny was constructed using the neighbor-joining method in MEGA7 software (Figure 1). As shown in Figure 1, the P450 protein sequence is divided into four different subfamilies. The group marked in red is the largest subfamily, which contains 81 P450 genes. Genes from the same subgroup can be considered to have the same function. P450 proteins derived from homologous chromosomes A subgroups and D subgroups tend to aggregate in the same branch. The conserved motifs in the P450 proteins were analyzed by MEME and TBtools software (Figure 2). The P450 gene is a family that contains a total of eight conserved motifs. The motifs contained in different genes were the same, indicating the conservatism of the motif domain for almost all the identified P450 family genes. The overall expression pattern of the first subgroup of motifs was 3_5_2_6_1_8; the expression pattern of the second subgroup was 2_6_7_8; the expression pattern of the third subgroup was 3_2_6_1_8; and the expression pattern of the fourth subgroup was 3_2_6_1_8.

### Gene Structure and *Cis-*Element Analysis of P450 Family Genes

**FIGURE 3:**
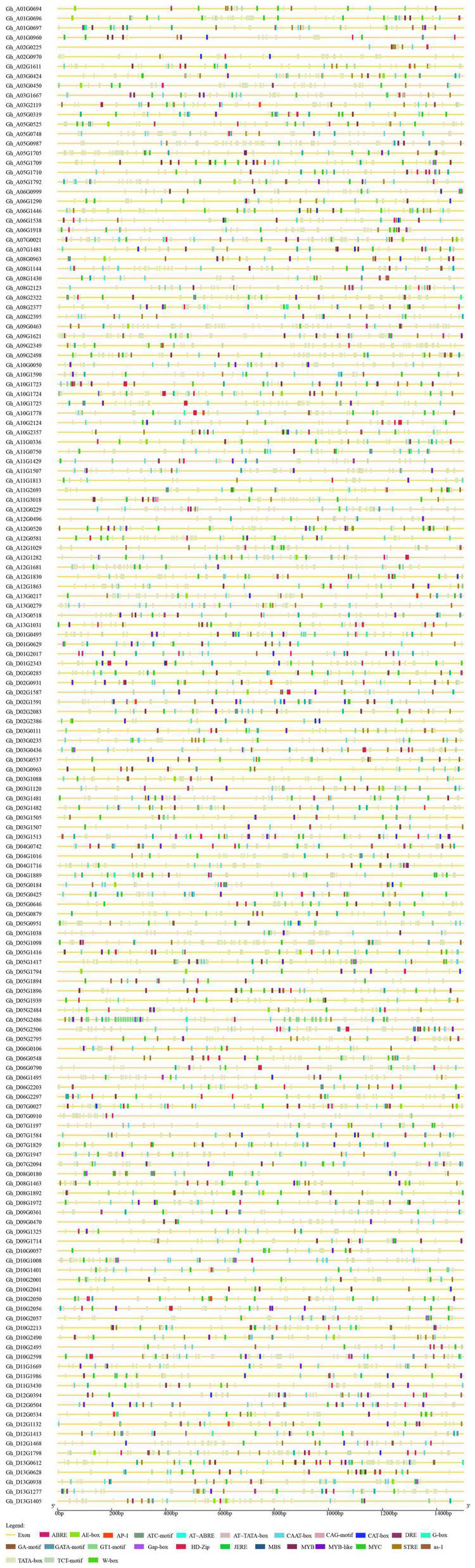
Upstream *cis-*acting elements of the P450 family genes in upland cotton.

The length of P450 genes in upland cotton differs greatly, ranging from 1,500 to 13,000 bp. The number of exons in the P450 family genes ranged from 1 to 9, while the number of introns ranged from 0 to 8, and there were similar trends within clusters (Figure 2). These results further confirmed the reliability of the phylogenetic tree. A total of 31 *cis-*acting elements were identified by analyzing a 1,500 bp promoter sequence (Figure 3), and differences existed in the *cis-*acting elements of these genes (Table 2).

**TABLE 2:** Annotation of *cis*-acting elements at the 5′ end of P450 gene.

Regulatory element	Core sequence	Element function
AAGAA-motif	GAAAGAA	Conserved sequence
ABRE	ACGTG	*Cis-*acting element involved in the abscisic acid responsiveness
AE-box	AGAAACTT	Part of a module for light response
AP-1	TGAGTTAG	Conserved sequence
ATCT-motif	AATCTAATCC	Part of a conserved DNA module involved in light responsiveness
ABRE	ACGTG	*Cis-*acting element involved in the abscisic acid responsiveness
AT∼TATA-box	TATATA	Conserved sequence
CAAT-box	CCAAT	Common *cis-*acting element in promoter and enhancer regions
CAG-motif	CAG-motif	Part of a light response element
CTAG-motif	ACTAGCAGAA	
CAT-box	GCCACT	*Cis-*acting regulatory element related to meristem expression
DRE		
MYC	CATGTG	
TATA	TATAAAAT	
G-box	TACGTG	*Cis-*acting regulatory element involved in light responsiveness
GA-motif	ATAGATAA	Part of a light responsive element
GATA-motif	AAGGATAAGG	Part of a light responsive element
GT1-motif	GGTTAA	Light responsive element
Gap-box	CAAATGAA(A/G)A	Part of a light responsive element
HD-Zip 1	CAAT(A/T)ATTG	Element involved in differentiation of the palisade mesophyll cells
MBS	CAACTG	MYB binding site involved in drought-inducibility
Myb	CAACTG	
MYB-like	TAACCA	
STRE	AGGGG	
Box II	ACACGTAGA	Part of a light responsive element
TATA-box	TATATTTATATTT	Core promoter element around -30 of transcription start
TCT-motif	TCTTAC	Part of a light responsive element
W box	TTGACC	
as-1	TGACG	
box S	AGCCACC	

### Chromosomal Location of the P450 Gene Family in Upland Cotton

**FIGURE 4:**
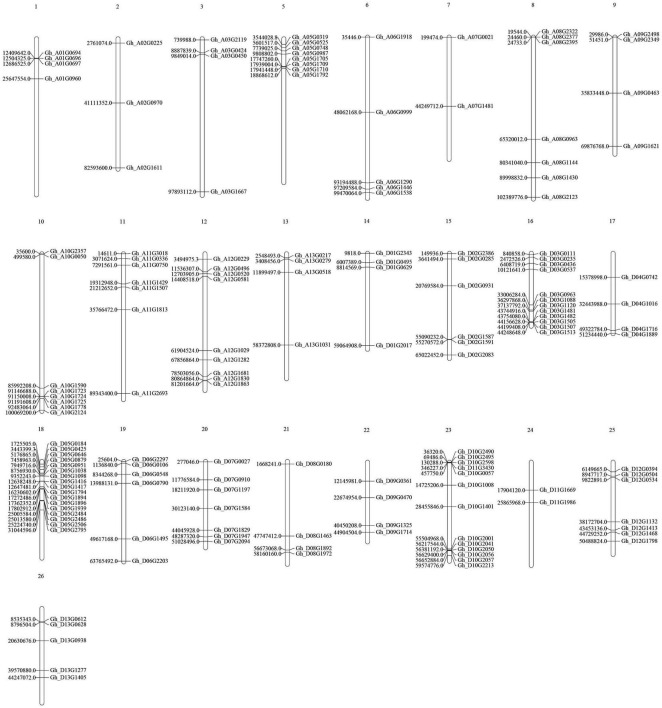
Locations of the P450 genes on upland cotton chromosomes.

The 156 genes were randomly distributed on 25 chromosomes of the cotton genome (Figure 4), and the number of genes varied on each chromosome. Chromosome D05, which contained 17 genes, had the largest number of genes. There is no P450 gene on Chromosome A04.

### Collinearity Analysis Within the Cotton Genome

**FIGURE 5:**
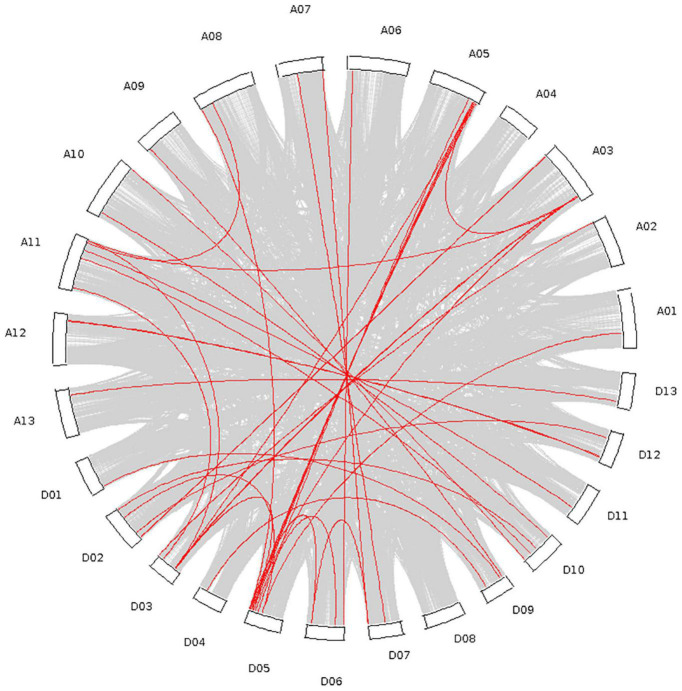
Collinearity analysis of the whole upland cotton genome and P450 family genes.

A total of 40 tandem repeat genes were submitted to gene collinearity analysis in the upland cotton genome (Supplementary Table 2), and the results are shown in Figure 5. There were many double duplications of genes among the AD subgroups. Furthermore, four genes on chromosomes A05 and D05 were duplicated.

### Analysis of Gene Expression for P450 Family Genes in Upland Cotton

**FIGURE 6:**
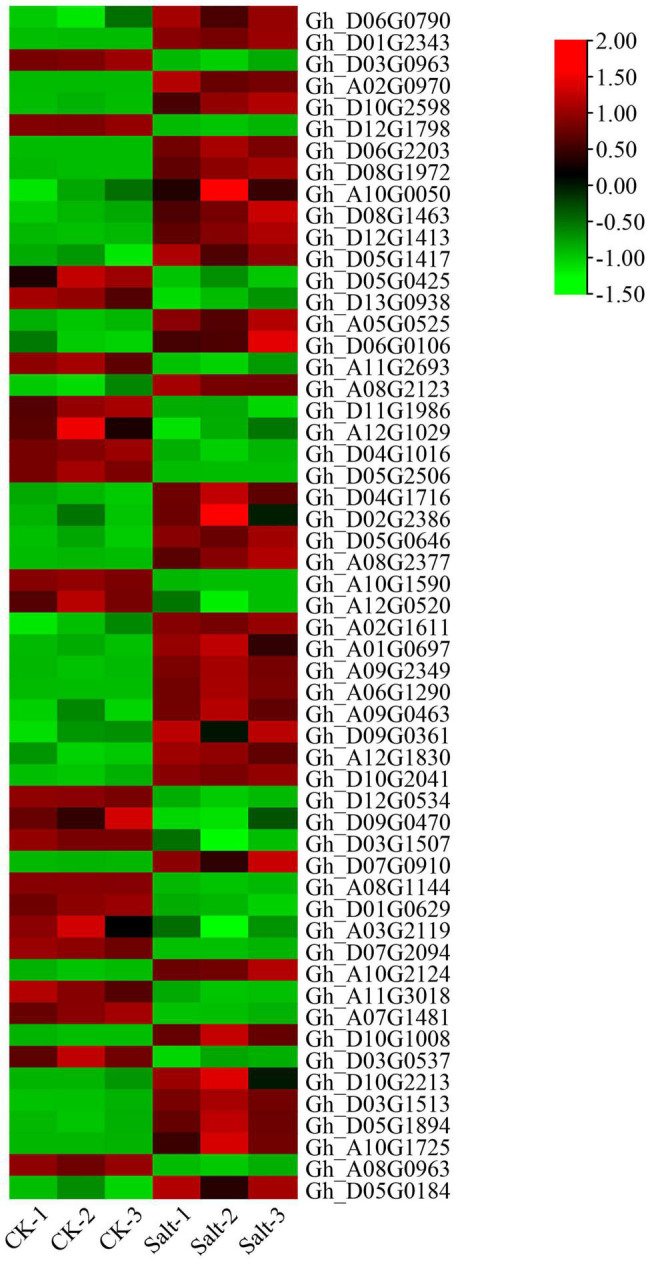
Expression patterns of the P450 family genes in the salt-tolerant transcriptome of upland cotton “Tongyan No. 1”.

**FIGURE 7:**
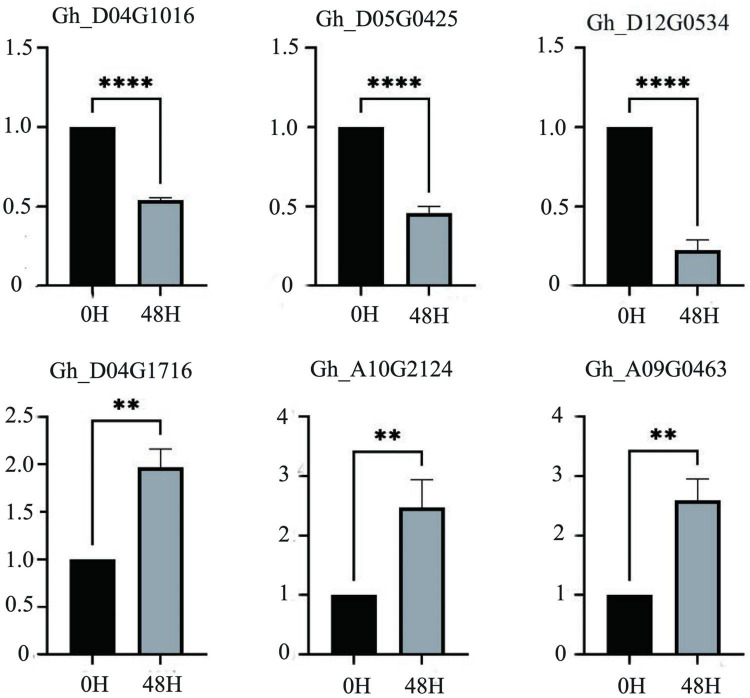
The relative expression levels of six candidate genes in upland cotton under salt stress. A asterisk on the bars indicate significant difference at 0.05 probability level by *t* test. **P ≤ 0.01, ****P ≤ 0.0001 in *t*-test.

According to the salt-tolerant transcriptome data of upland cotton obtained in a previous study, 55 differentially expressed P450 family genes belonging to different subfamilies were identified (Figure 6). We selected six DEGs out of these 55, three genes with significantly upregulated expression and three genes with significantly downregulated expression of transcription, to perform qRT-PCR verification experiments. The expression of the three genes, *Gh_D04G1016*, *Gh_D05G0425* and *Gh_D12G0534*, was significantly downregulated after 48 h in the salt treatment, whereas the expression of the remaining three genes, *Gh_A09G0463*, *Gh_D04G1716* and *Gh_A10G2124*, was significantly upregulated after 48 h of treatment. The results of the qRT-PCR were consistent with the expression trends in the transcriptome analysis (Figure 7 and Supplementary Table 3).

## Discussion

Soil salinization seriously reduces crop yields. As cotton is one of the most important crops in the world, it is particularly important to develop salt-tolerant cotton varieties and rationally utilize saline-alkali land. With the release of the genome sequencing of different cotton species (Li et al., 2015; Zhang et al., 2015; Udall et al., 2019; Chen et al., 2020; Huang et al., 2020), it is possible to obtain the sequence and structure of gene families and compare the evolutionary relationship of different genes at the genome level.

According to the transcriptome data of the salt-tolerant upland cotton line “Tongyan No. 1” obtained in our previous study, P450 family genes have significant differential expression under salt stress, and according to the Kyoto Encyclopedia of Genes and Genomes (KEGG), a large number of genes are enriched in pathways related to sesquiterpenes and triterpenes. Cotton has a large amount of gossypol and related sesquiterpenoids and is a unique polyphenolic compound. The pigment glands of cotton are known to be support pest resistance and enable strong adaptability of cotton under adverse conditions. Sesquiterpenoids also serve as plant protection elements, resist pathogens, and prevent animals from consuming plants. Moreover, they can also be used as a signal molecule, participating in the interaction between organisms. Sesquiterpenoids are highly diverse and are closely related to the diversity of enzyme genes in secondary metabolic pathways, including P450. At present, there are relatively few studies on the function of the P450 gene family. According to the published genome information of the standard genetic line of upland cotton (TM-1), 156 protein sequences containing complete P450-specific domains were identified by BLASTP, and their molecular weights were 5,949.6–245,576.3 Da, while the corresponding number of encoded amino acids was 51–2,144. The P450 family genes encode proteins with different physical and chemical properties, with obvious differences in size and different functions and regulatory mechanisms, whereas they all possess a stable P450 domain.

Through analysis of the phylogenetic tree, the P450 family genes of the upland cotton species were divided into four subgroups. In the analysis of the structure of the P450 family genes and the conserved motifs, the four subgroups had similar gene structures and conserved sequence expression. Moreover, the existence of a large number of genes in the four subgroups also proves that the P450 family has an important and stable function in the genetic development of upland cotton. The analysis of collinearity within the genome also proved that there are a large number of tandem duplication effects between homologous chromosomes. Duplication is the most common means of amplification of gene families (Cannon et al., 2004). The presence of multiple gene copies can prevent the loss of function caused by genetic mutations, suggesting the importance of gene function. The large amount of duplication allows the P450 family to participate in a variety of stress resistance pathways and phytoalexin production. The degree of conservation in the P450 gene family is very high. The 156 genes are located and distributed on 25 chromosomes of cotton. Except for Chromosome A04, all the chromosomes have P450 family genes. Most of the genes are distributed in the two ends and the middle of the chromosome, with stable heritability, which also shows the importance and widespread nature of the P450 family.

By identifying the upstream *cis-*elements of each gene, it is possible to better study the regulatory mechanism of each gene. P450 family genes contain several *cis-*elements. These *cis-*elements do not encode proteins but can regulate gene expression. Differences exist in *cis-*acting elements in genes (Figure 3), which means that each gene has different regulatory mechanisms (Table 2). The existence of *cis-*elements increased the complexity of the gene-encoded protein, and the function of *cis-*acting elements needs further research. When comparing the P450 family genes we obtained with the salt-tolerant transcriptome data of upland cotton, 55 differentially expressed P450 family genes were obtained. These 55 genes have significant differential expression under salt stress, and they are used as the target genes of the P450 family participating in salt stress regulation.

We randomly selected and verified six of these genes by qRT-PCR according to the up- and downregulation of gene expression (Figure 7). The consistency between the results of the qRT-PCR and transcriptome analysis indicated that these genes were likely to be involved in the salt tolerance of the cotton seedlings. Among the downregulated genes, *Gh_D04G1016* is the *CYP76A2* gene. The CYP76 subfamily improves stress tolerance in Arabidopsis, can decompose toxic substances in plants, and participates in redox reactions in plants. After salt treatment, *CYP76A2* was greatly downregulated (Siminszky, 2006; Matthes et al., 2011; Wang et al., 2012; Ilc et al., 2018). *Gh_D05G0425* is the *CYP711A1* gene, which is involved in the synthesis of strigolactone. Strigolactone is a new type of hormone. It was first discovered in cotton and has the effect of resisting salt stress and drought stress. After 48 h of salt stress, the expression of Gh_D05G0425 was significantly down-regulated (Benveniste et al., 2006; Wakabayashi et al., 2020). *Gh_D12G0534* is the *CYP81D1* gene. The *CYP81D1* gene is considered to be a suitable marker gene for studying transcriptional activation mechanisms under chemical stress. It shares a common expression and synthesis pathway with jasmonic acid, which can induce plants to excrete harmful substances. We speculate that the synthesis of jasmonic acid is increased under salt stress to resist salt damage, so CYP81D11 gene expression is down-regulated (Didierjean et al., 2002; Siminszky, 2006; Matthes et al., 2011). Among the genes with upregulated expression levels, the function of *Gh_A09G0463* is currently unknown, and the specific pathway needs further study. *Gh_D04G1716* is the *CYP82C4* gene, which is related to Fe ion transport. Because salt stress causes an imbalance of ions inside and outside the plant to increase iron ion transport, the expression of this gene is upregulated after salt treatment (Murgia et al., 2011; Ilc et al., 2018). *Gh_A10G2124* is a *CYP71A1* gene that is involved in the synthesis of indole acetic acid, and indole acetic acid can promote the growth of plants. Under salt stress, plants increase the expression of this gene to control the synthesis of indole acetic acid and promote growth (Wang et al., 2012). Regarding other genes in the P450 gene family, it was found that *Gh_A08G0963* affected the size of the seed (Meng et al., 2015); *Gh D13G0938* was identified for herbicide resistance; *Gh A06G1290* and *Gh_D10G2041* led to male sterility in cotton; *Gh_D06G0790* regulated ROS activity and promoted auxin signaling pathway to enhance resilience by activating T31B5_170 expression; *Gh_D09G0361* participated in two successive oxidation steps in the synthesis of the plant hormone jasmonoyl isoleucine, enabling it to promote catabolism. After all, P450 family has many genes, and the functions of unknown genes in the same family can be inferred through known genes. It is speculated that under salt stress, the P450 family genes enhanced the adaptability of cotton by regulating gossypol and sesquiterpenoids. However, the functions and regulatory mechanisms of individual genes need further research. The expression of the P450 family under other abiotic stresses also needs further study. The identification and analysis of the P450 gene family presented in this article is helpful for breeding salt-tolerant cotton varieties and provides a basis for the study of cotton salt stress response pathways.

## Data Availability Statement

The datasets presented in this study can be found in online repositories. The names of the repository/repositories and accession number(s) can be found in the article/Supplementary Material.

## Author Contributions

KS, HF, and YC performed most of the experiments and data analysis. ZZ, QC, and TS helped in sample preparation. MK and JZ helped design the experiments and revise the manuscript. BW designed the experiments and edited the manuscript. All authors read and approved the final manuscript.

## Conflict of Interest

The authors declare that the research was conducted in the absence of any commercial or financial relationships that could be construed as a potential conflict of interest.

## Publisher’s Note

All claims expressed in this article are solely those of the authors and do not necessarily represent those of their affiliated organizations, or those of the publisher, the editors and the reviewers. Any product that may be evaluated in this article, or claim that may be made by its manufacturer, is not guaranteed or endorsed by the publisher.

## Footnotes

1
http://mascotton.njau.edu.cn/Data.htm

2
http://pfam.xfam.org/

3
http://smart.embl-heidelberg.de

4
http://www.NCBI.nlm.NIH.gov/Structure/cdd/wrpsb

5
http://www.expasy.org

6
http://www.omicsclass.com/article/671

7
http://meme-suite.org/

8
http://gsds.cbi.pku.edu.cn/

9
http://bioinformatics.psb.ugent.be/webtools/plantcare/html/

10
http://www.heatmapper.ca/expression/
